# Stab Injury of the Thoracic Aorta: Computed Tomography Findings

**DOI:** 10.1155/2013/397514

**Published:** 2013-02-06

**Authors:** Seyma Yildiz, Huseyin Toprak, Asli Serter, Ercan Kocakoç

**Affiliations:** Department of Radiology, Faculty of Medicine, Bezmialem Vakif University, Fatih, 34090 Istanbul, Turkey

## Abstract

Stab injury of the thoracic aorta is a rare condition with high mortality rate. Patients must be evaluated carefully, and the diagnosis usually should be confirmed by radiological modalities. In this case, we report a 37-year-old man presented with a penetrating stab injury to the upper back and the thoracic aorta, and the diagnostic role of computed tomography is discussed.

## 1. Introduction

Thoracic traumas take the third place after head and extremity traumas in patients with trauma who presented to emergency department. Thoracic traumas cause the 20–25% of deaths due to trauma in the first four decades of life [1]. Thoracic traumas are caused by blunt and penetrating injuries. Stab injuries of the thoracic aorta are rare. Because the thorax contains the heart, lungs, and great vessels, any thoracic injury of this space is associated with a high mortality rate. In thoracic traumas, accurate diagnosis and appropriate surgical approach form the basis of reducing the morbidity and mortality in such injuries. In this case we report a patient who was admitted to our emergency department due to stab injuries in the upper back region, and diagnostic tools of computed tomography (CT) are discussed.

## 2. Case Report

**Figure 1:**
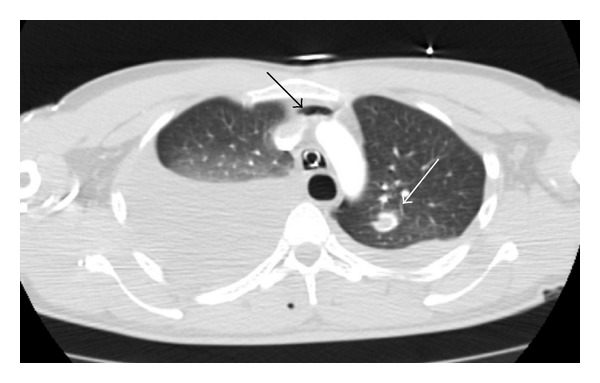
Contrast-enhanced axial chest CT using the lung window settings reveals bilateral hemothorax, minimal pneumothorax, and intraparenchymal hematoma in left lower laterobasal segment.

**Figure 2:**
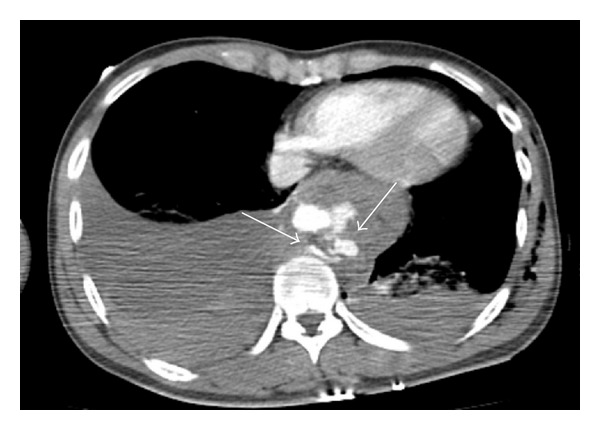
Contrast-enhanced axial chest CT using the mediastinal window settings shows hematoma surrounding trachea posteriorly and aorta circumferentially in the posterior mediastinum.

**Figure 3:**
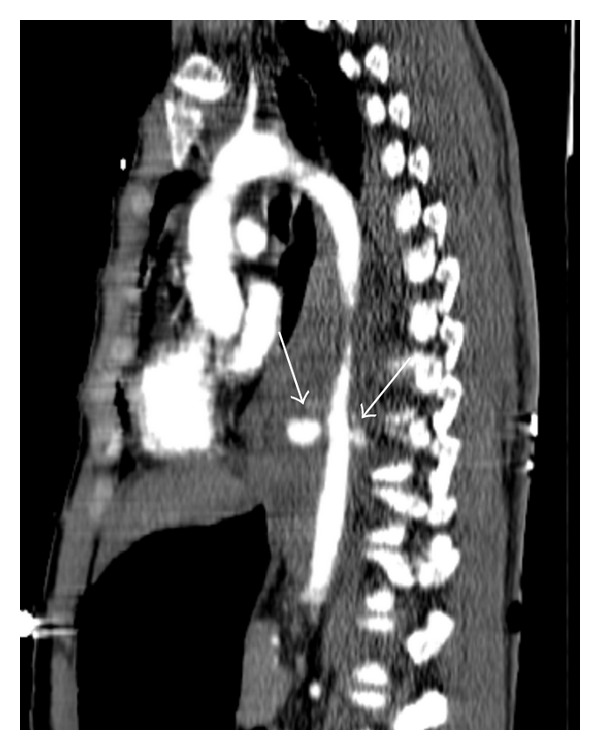
Contrast-enhanced sagittal chest CT using the mediastinal window settings demonstrates active contrast material leak from the hematoma located in the posterior mediastinum, and this is considered as aortic rupture.

A 37-year-old unresponsive man with penetrating injuries was admitted to our emergency department. On his physical examination, pulse and blood pressure could not be retrieved. Glasgow coma scale was determined as 3. There were 3 cm long bleeding laceration at the infrascapular region and midline 4 cm long bleeding laceration at the lumbar region. He was accepted as a respiratory and cardiac arrest; he was resuscitated and he responded to resuscitation. During evaluation of thorax, minimal pneumothorax was revealed, a chest tube was inserted, and 4 units of erythrocyte suspension were given to the patient. In order to evaluate the intrathoracic lesions, a contrast-enhanced CT was performed. Thorax CT examination revealed bilateral high density fluid collections at pleural space which were considered as hemothorax, left-sided pneumothorax, and an intraparenchymal hematoma in left lower laterobasal segment of the lung (Figure 1). Soft-tissue density hematoma that was surrounding trachea posteriorly and aorta circumferentially was seen in posterior mediastinum (Figure 2). From this area, adjacent to descending thoracic aorta, active contrast material leak was seen, and therefore this was considered as aortic rupture and contrast material extravasation due to acute aortic rupture (Figure 3). The patient underwent an operation by thoracic and cardiovascular surgeons. During operation, active bleeding could not be stopped and the patient died.

## 3. Discussion 

Thoracic aortic traumas have high mortality rate and in 80–90% of cases are fatal [2]. Mortality in aortic trauma depends on several factors such as severity of trauma, transit time to trauma center, and hemodynamic stability at admission [3]. Penetrating cardiac injuries can be difficult to diagnose, and sometimes diagnosis may be missed. 14% of cases die before a diagnosis can be made [4].

Large vascular structures are usually located at the posterior part of the thorax. Therefore, in any penetrating injury from the posterior part of the thorax, due to the higher risk of injury to these vascular structures, the risk of death is higher. Small caliber injury to the great vessels such as aorta, superior vena cava, and inferior vena cava can cause significant blood loss. In our case, soft-tissue density hematoma, surrounding trachea posteriorly and aorta circumferentially, was seen in posterior mediastinum. From this hematoma, adjacent to descending thoracic aorta, active contrast material leak was seen, and therefore this was considered as acute aortic rupture and contrast material extravasation due to aortic rupture.

In most of the cases who can reach the emergency surgical unit, there is hemodynamic disturbance. In cases with thoracic aortic injury admitted to the emergency clinics on foot, clinical signs and symptoms are nonspecific. In the majority of these cases, no clinical finding is seen until the hemodynamic instability develops [5]. Symptoms related with thoracic aortic injury are caused by tension in mediastinal supportive tissue developed secondary to mediastinal hemorrhage. The major symptoms are retrosternal pain, interscapular pain, dyspnea, hoarseness, and cough [5]. Hemothorax and pneumothorax due to lung parenchymal injury, absence of pulse due to great artery injury, signs of tamponade due to pericardial effusion, and neurologic deficits such as paraplegia may be seen.

Chest radiography is often used as a first-line diagnostic modality. On chest radiography, massive hemothorax or pneumothorax developed secondary to trauma which may be fatal can be excluded. In addition, on chest radiography, mediastinal widening, displacement of mediastinal structures, and depression of left main bronchus which may occur secondary to bleeding from aorta may also be seen. Rib fractures associated with aortic injury may also be identified with chest X-ray [3]. The diagnostic sensitivity of CT is approximately 98% [6]. On CT, if the fat plane between aorta and hematoma could not be demarcated, it can be said that the bleeding source is aorta. Rupture, intimal flap, traumatic pseudoaneurysm, intraluminal mural thrombus, and aortic contour abnormalities are direct findings of aortic injury [7]. If the fat plane around thoracic aorta is preserved, the bleeding source is not aorta.

In our case, CT revealed bilateral high density fluid collections at pleural space which were considered as hemothorax, left-sided pneumothorax, and an intraparenchymal hematoma in left lower laterobasal segment. Adjacent to thoracic aorta, active contrast leakage suggestive of aortic rupture was seen.

Conventional angiography is considered as a gold standard examination method in the evaluation of aortic injury; sensitivity of angiography is approximately 100%, specificity is approximately 98%, and accuracy is higher than 99% [8]. Findings range from vague contour irregularities of aorta to contrast material extravasation can be seen. In addition, findings secondary to aortic injury such as pseudoaneurysm, intraluminal filling defect, and intraluminal irregularities may be seen [5]. In cases in which the aortic injury can be diagnosed with CT, due to hyperacute situation of the aortic injury, it should be noted that angiography spends the patient's precious time.

In conclusion, in patients with thoracic trauma, especially with stab injuries, patients must undergo a CT examination to exclude major vessel injury. CT examination helps us in accurate diagnosis; therefore appropriate surgical approach can be performed in order to reduce the morbidity and mortality.
